# Excitation of coherent propagating spin waves by pure spin currents

**DOI:** 10.1038/ncomms10446

**Published:** 2016-01-28

**Authors:** Vladislav E. Demidov, Sergei Urazhdin, Ronghua Liu, Boris Divinskiy, Andrey Telegin, Sergej O. Demokritov

**Affiliations:** 1 Institute for Applied Physics and Center for Nanotechnology, University of Muenster, Correnstrasse 2-4, Muenster 48149, Germany; 2 Department of Physics, Emory University, Atlanta, Georgia 30322, USA; 3 M.N. Miheev Institute of Metal Physics of Ural Branch of Russian Academy of Sciences, Yekaterinburg 620041, Russia

## Abstract

Utilization of pure spin currents not accompanied by the flow of electrical charge provides unprecedented opportunities for the emerging technologies based on the electron's spin degree of freedom, such as spintronics and magnonics. It was recently shown that pure spin currents can be used to excite coherent magnetization dynamics in magnetic nanostructures. However, because of the intrinsic nonlinear self-localization effects, magnetic auto-oscillations in the demonstrated devices were spatially confined, preventing their applications as sources of propagating spin waves in magnonic circuits using these waves as signal carriers. Here, we experimentally demonstrate efficient excitation and directional propagation of coherent spin waves generated by pure spin current. We show that this can be achieved by using the nonlocal spin injection mechanism, which enables flexible design of magnetic nanosystems and allows one to efficiently control their dynamic characteristics.

## Abstract (web summary)

Dynamical effects driven by pure spin currents in magnetic nanostructures have so far been limited to spatially confined excitations, limiting potential magnonic applications. Here, the authors demonstrate the generation of coherent propagating spin waves in NiFe films induced by pure spin currents.

## Graphical abstract

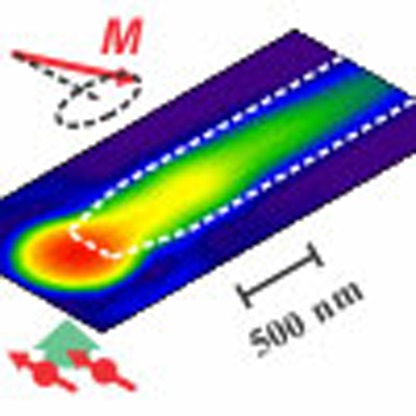

Recent intense studies of the interactions of magnetization with pure spin currents, produced either by the spin Hall effect (SHE)^1^,^2^,^3^ in materials with strong spin–orbit interaction or by the nonlocal spin injection (NLSI)^4^,^5^,^6^, have resulted in the development of novel nanodevices that use spin currents to induce coherent magnetization oscillations^7^,^8^,^9^,^10^,^11^,^12^,^13^,^14^,^15^,^16^,^17^. As SHE and NLSI do not require the flow of electrical current through the active magnetic layer, their utilization allows one to reduce Joule power dissipation in magnetic nanodevices and provides the possibility to use insulating magnetic materials^18^. The lack of the requirement for the electrical current flow through the active magnetic layers also results in a unique flexibility compatible with a variety of novel magnetic nano-oscillator geometries including nanowires^12^, nanoconstrictions^11^, nanogaps^7^,^9^,^10^,^13^,^14^,^16^,^17^ and nanocontacts^15^. This flexibility enables straightforward adaptation of devices operated by spin current to the needs of specific applications and their incorporation as building blocks in integrated electronic circuits.

The possibility to control magnetization dynamics by pure spin currents is especially attractive for magnonics^19^,^20^,^21^,^22^,^23^, which uses propagating spin waves as the nanoscale signal carrier. Possible applications of spin current-induced phenomena in magnonic nanocircuits include amplification, manipulation, and excitation of spin waves. The latter can have a particularly significant impact on the development of magnonics, as the traditional inductive method becomes very inefficient on the nanoscale^22^. Nevertheless, excitation of coherent propagating spin waves by pure spin currents has not yet been achieved, owing to a number of conflicting requirements, which are also known for the traditional devices operated by spin-polarized electric currents^24^,^25^,^26^,^27^,^28^,^29^. In particular, efficient excitation of single-frequency coherent magnetization oscillations generally requires that they are spatially confined, as was shown for all the demonstrated nano-oscillators operated by pure spin currents^7^,^8^,^9^,^10^,^11^,^12^,^13^,^14^,^15^,^16^,^17^. Equivalently, the dynamical mode that enters the auto-oscillation regime under the influence of spin current does not radiate energy in the form of propagating spin waves. Moreover, as the spin torque effect (STT)^30^,^31^ underlying the spin current-induced dynamics is exerted only at the magnetic interfaces, maximizing the effect of spin current requires that the thickness of the active magnetic layer does not exceed a few nanometres. However, spin waves rapidly decay in thin magnetic films. Thus, relatively thick active magnetic layers must be used to achieve propagation length of several micrometres acceptable for integrated magnonic circuits^19^,^20^,^21^,^22^,^23^.

Here, we experimentally demonstrate a spin current-driven nanomagnonic system that simultaneously satisfies the conflicting requirements described above. This is accomplished by hybridizing two magnetic subsystems with different dynamic characteristics: the active subsystem in which a spatially confined dynamical mode is excited by the spin current and the spin-wave guiding subsystem that facilitates efficient propagation of spin waves. We show that the NLSI spin-current generation mechanism provides the geometric flexibility required for such a hybrid magnetic structure. The demonstrated system exhibits efficient and controllable excitation and directional propagation of coherent spin waves characterized by a large decay length.

## Results

### Test devices

**Figure 1:**
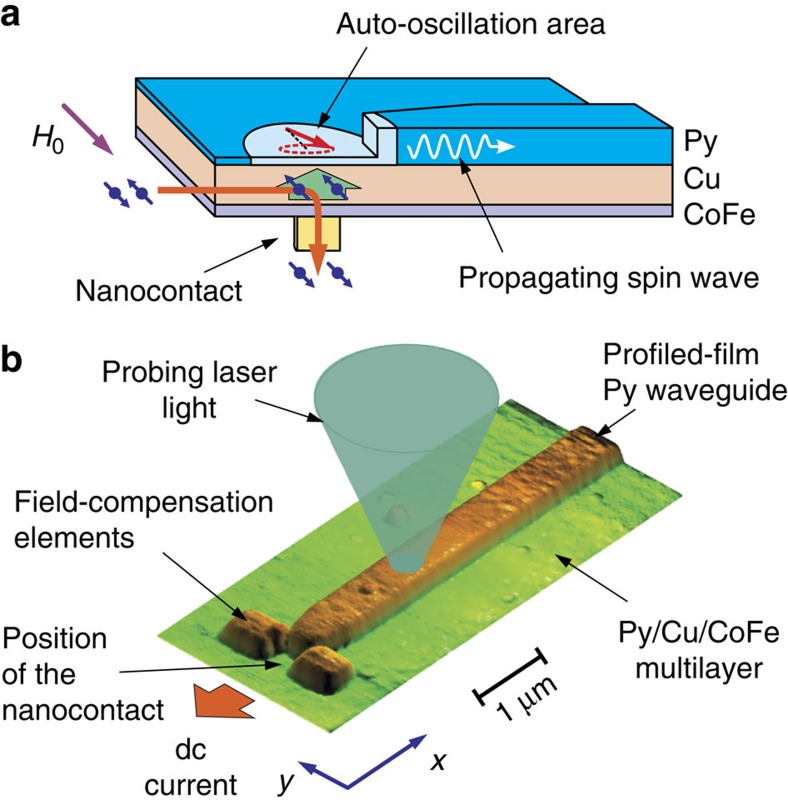
Experimental layout. (**a**) Schematic of the test device. The device consists of a 5-nm-thick Py active magnetic film separated from the 8-nm-thick CoFe spin injector by a 20-nm-thick layer of Cu. A 20-nm-thick and 500-nm-wide Py spin-wave waveguide is fabricated on the surface of the extended Py(5) film. The electric current is injected into the multilayer through a 60-nm-circular nanocontact fabricated on the CoFe side. The red arrow shows the corresponding flow of electrons. The green arrow shows the spin current generated due to the spin accumulation in Cu above the nanocontact. The device is magnetized by the static magnetic field *H*_0_. (**b**) AFM image of the device topography. The height of the waveguiding structure is 20 nm. The waveguiding strip is tapered to the width of 300 nm at the edge, providing a uniform distribution of the internal static magnetic field throughout the entire waveguide. Two additional rectangular Py(20) elements with dimensions of 500 nm by 300 and 500 nm edge-to-edge separation are fabricated beside the nano-oscillator, to minimize the effects of the dipolar fields.

The schematic of our experiment is shown in Fig. 1a. The studied device consists of a 5-nm-thick Permalloy (Py) active magnetic film separated from the 8-nm-thick CoFe spin injector by a 20-nm-thick layer of Cu. The electric current is injected into the multilayer through a 60-nm circular nanocontact fabricated on the CoFe side. Away from the contact area, ∼88% of the injected current is drained through the Cu film, whereas the remaining 12% is distributed between CoFe and Py layers. We note that the current vanishes in Py right above the nanocontact, because of the cylindrical symmetry of the system (see Supplementary Fig. 1 for the detailed analysis of the current flow). The red arrow in Fig. 1a shows the corresponding flow of electrons. The injected electrons become spin polarized due to the spin-dependent scattering in CoFe and at the Cu/CoFe interface^32^, resulting in spin accumulation in Cu above the nanocontact. Spin diffusion away from this region produces a spin current flowing into the Py layer, exerting STT on its magnetization. The magnetizations of both CoFe and Py layers are aligned with the saturating static in-plane magnetic field *H*_0_. For positive driving electric currents, as defined by red arrows in Fig. 1a,b, the magnetic moment carried by the spin current is antiparallel to the magnetization of the Py layer, resulting in the STT compensating the dynamic magnetic damping. When damping is completely compensated by the spin current, the magnetization of the Py layer exhibits auto-oscillations in the spatial area with the size of ∼300 –400 nm determined by the spin current injection region^15^.

To convert these localized magnetization oscillations into a propagating spin wave, a 20-nm-thick and 500-nm-wide Py strip aligned perpendicular to the direction of *H*_0_ is fabricated on the surface of the extended Py(5) film. The waveguide is terminated at a distance of 150 nm from the centre of the nanocontact. This distance is sufficiently small to ensure efficient dynamic coupling between the current-induced magnetic auto-oscillations in the thin film and the magnetization in the strip. In contrast to the thin Py(5) film, the increased thickness strip supports propagating spin waves at the frequency of auto-oscillations, which are characterized by a large decay length. Thus, the energy of the confined oscillations can be radiated and directionally guided by the strip that plays the role of a magnonic nano waveguide^33^.

Atomic-force microscopy image of the studied device (Fig. 1b) shows additional details of the studied structure. The waveguiding strip is tapered to the width of 300 nm at the edge, providing a uniform distribution of the internal static magnetic field throughout the entire waveguide. The dipolar field of the strip at the location of the auto-oscillator can downshift the auto-oscillation frequency, resulting in a frequency mismatch with the propagating spin waves in the strip. To minimize this effect, two additional rectangular Py(20) elements with dimensions of 500 nm by 300 and 500 nm edge-to-edge separation are fabricated beside the nano-oscillator (see Supplementary Fig. 2 for details).

### Magnetooptical measurements

To detect the current-induced magnetization dynamics, we use the micro-focus Brillouin light scattering (BLS) spectroscopy^34^. We focus the probing laser light on the surface of the magnetic film into a diffraction-limited spot (Fig. 1b) and analyse the spectrum of light inelastically scattered from the magnetic oscillations. The resulting BLS signal is proportional to the local intensity of the oscillations at the selected frequency.

**Figure 2:**
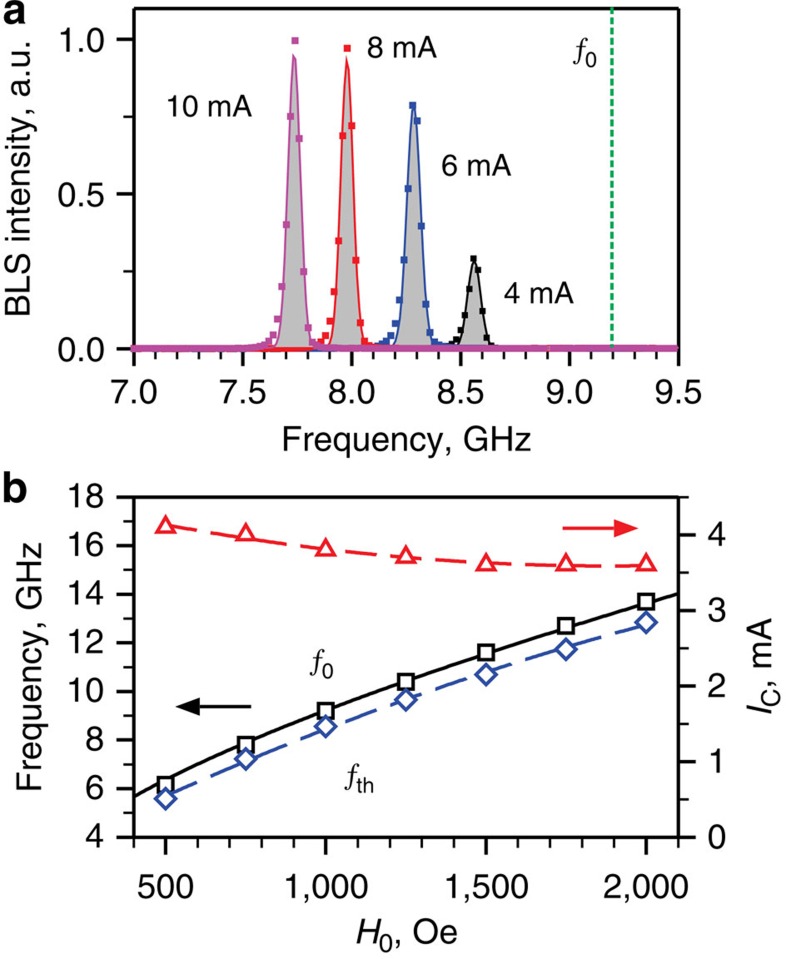
Spectral characteristics of the spin-current nano-oscillator. (**a**) BLS spectra of the current-induced magnetization auto-oscillations at different driving currents, as labelled. Vertical dashed line marks the frequency *f*_0_ of the uniform ferromagnetic resonance (FMR) in the Py film. The data were recorded at *H*_0_=1,000 Oe. The linewidth is determined by the limited spectral resolution of the measurement apparatus. (**b**) Static-field dependences of the FMR frequency *f*_0_ (squares), the auto-oscillation frequency at the onset current (diamonds) and the auto-oscillation onset current (triangles). Solid curve is the fit of the experimental data by the Kittel formula. Dashed curves are the guides for the eye.

To characterize the operation of the NLSI nano-oscillator, the probing laser spot was located directly at the position of the nanocontact. Figure 2a shows the spectra of magnetic oscillations detected by BLS at different driving currents. The onset of auto-oscillations is signified by a narrow intense spectral peak that emerges at currents above *I*_C_=3.6 mA. With increasing *I*>*I*_C_, the intensity of the auto-oscillation peak gradually increases, whereas its frequency decreases due to the nonlinear frequency shift. We emphasize that the auto-oscillation frequency is below the frequency *f*_0_ of the uniform ferromagnetic resonance in the extended Py film determined from independent BLS measurements at *I*=0 (dashed curve in Fig. 2a). Thus, there are no propagating spin-wave states available in the Py film at the frequency of auto-oscillation, and therefore the oscillation is spatially localized and does not radiate spin waves into the surrounding film. Similar behaviors are observed in a broad range of applied fields from 500 to 2,000 Oe (Fig. 2b). The field-dependent data show that the auto-oscillation frequency is easily tunable by the static field, with minimal effect on the onset current that varies by no more than 10% over the entire field range. By comparing the data of Fig. 2 with those obtained for standalone NLSI oscillators^15^, we conclude that the oscillation characteristics are not adversely affected by the integration of the oscillator into the magnonic system.

**Figure 3:**
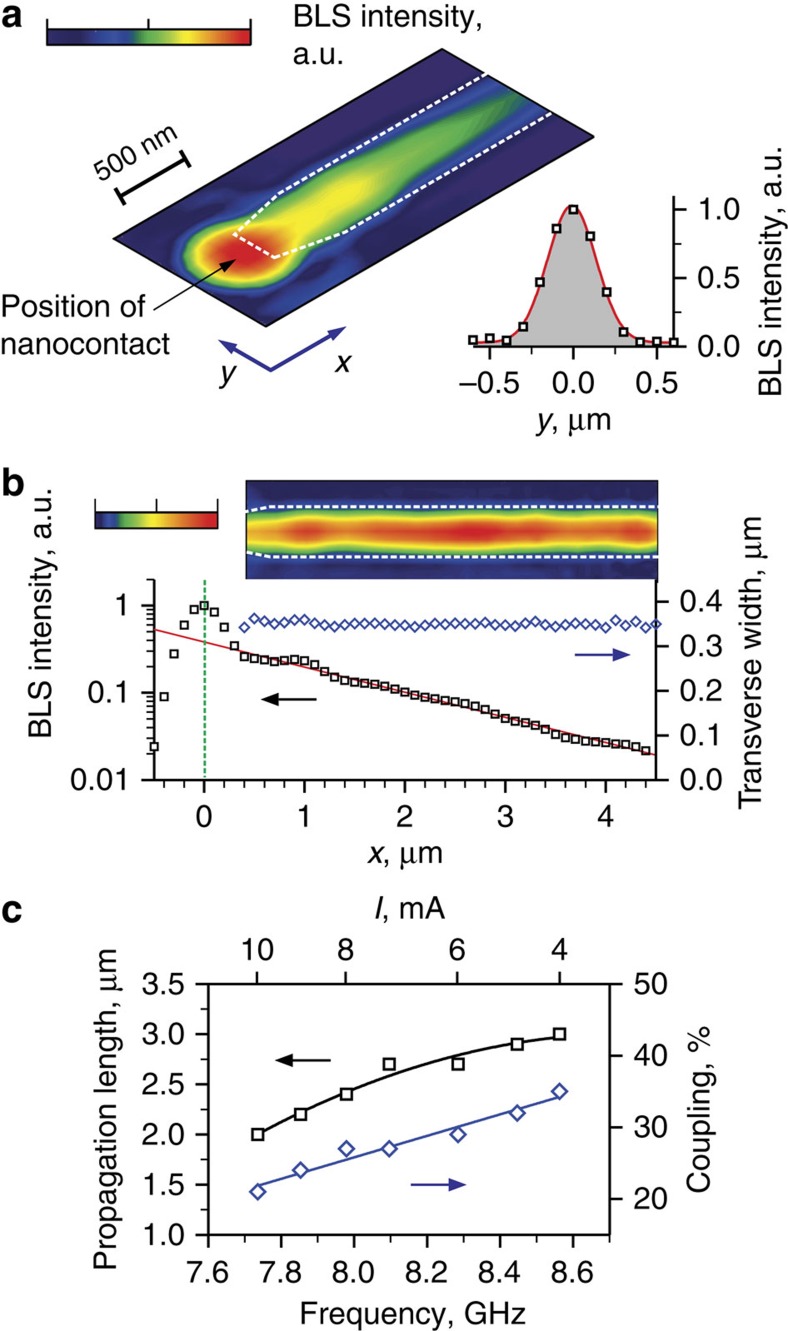
Propagation characteristics of the current-induced spin waves. (**a**) Normalized colour-coded map of the measured BLS intensity proportional to the time-averaged intensity of the dynamic magnetization. Colour scale: blue, 10^−2^; red, 1. Dashed line indicates the contour of the waveguide stripe. Inset: transverse section of the map at the distance of 2 μm from the nano-oscillator. Symbols are experimental data, line is the fit by the Gaussian function. (**b**) Propagation-coordinate dependence of the BLS intensity integrated across the transverse direction (squares) and that of the full width of the transverse profiles at half maximum (diamonds). Solid curve shows the results of the fit by the exponential function. Dashed vertical line shows the position of the nanocontact. Inset: decay-compensated map of the BLS intensity in the propagation area. Colour scale: blue, 0; red, 1. The data were recorded at *I*=4 mA and *H*_0_=1,000 Oe. (**c**) Dependences of the propagation length of current-induced spin waves and of the coupling coefficient between the NLSI nano-oscillator and the waveguide on the auto-oscillation frequency (driving current). Solid curves are the guides for the eye. The measurement uncertainty is smaller than the size of the symbols.

To investigate the spin wave emission by the nano-oscillator into the magnonic waveguide, spatial BLS intensity maps were recorded by rastering the probing laser spot, as shown in Fig. 3a for a 3 μm × 1.2 μm region encompassing the nano-oscillator and the adjacent area of the waveguide. This map characterizes the spatial distribution of the local intensity of the current-induced dynamic magnetization. It clearly shows two merged but distinct dynamical regions. The first circular high-intensity region is centred on the nanocontact. In this region, the magnetization oscillations are excited by the spin current. It is merged with another arrow-shaped increased-intensity region aligned with the strip waveguide, which is indicated by a dashed contour in Fig. 3a. The increased intensity is entirely confined to the waveguide, as shown by the transverse section of the intensity map, inset in Fig. 3a. These observations are consistent with the directional propagation of a spin wave excited in the waveguide by the spin current-induced oscillations.

To characterize the propagation characteristics of the excited spin wave, we analysed the dependence of BLS signals on the propagation coordinate *x*, which is defined as the distance from the nanocontact measured along the waveguide strip. Squares in Fig. 3b show the BLS intensity integrated across the transverse sections of the intensity map. These data plotted on the logarithmic vertical scale show that the spin wave exhibits a well-defined exponential decay ∼exp(−2*x*/*ξ*) along the waveguide. Here, *ξ* is the propagation length−the distance over which the spin wave amplitude decreases by a factor of *e* in the propagation direction. By fitting these data with the exponential function (red curve in Fig. 3b), we obtain *ξ*=3.0 μm, which is sufficiently large for the practical implementations of magnonic nanosystems^19^,^20^,^21^,^22^,^23^.

The BLS data also allow one to determine the efficiency of spin wave excitation in the waveguide, owing to the dynamical coupling to the nano-oscillator. We extrapolate the exponential spin-wave decay curve to the position *x*=150 nm corresponding to the edge of the waveguide and find the ratio between this value and the intensity at the position *x*=0 of the nanocontact, which characterizes the energy of the localized auto-oscillation mode. From the data of Fig. 3b, we obtain coupling of ∼35%, demonstrating the possibility to achieve a high efficiency for the proposed structure.

To visualize the spatial characteristics of the spin-wave beam over the entire propagation path, we can compensate for the exponential decay of the propagating spin wave by multiplying the experimental data by exp(2*x*/*ξ*). The compensated map (inset in Fig. 3b) clearly demonstrates that the spin wave is entirely concentrated in the waveguide and its energy is not lost to the radiation into the surrounding Py(5) film. This conclusion is confirmed by the quantitative analysis of the full width of the transverse spin-wave intensity profiles (diamonds in Fig. 3b). The absence of the dependence on the propagation coordinate indicates negligible spreading of the propagating spin-wave beam.

By performing similar measurements for different driving currents, we determined that both the spin wave propagation length and the coupling efficiency decrease with increasing *I* (Fig. 3c). As will be shown below, this decrease is associated with the decreasing frequency of auto-oscillation (bottom horizontal scale in Fig. 3c). Nevertheless, the decrease of both parameters is moderate, allowing one to tune the operating frequency not only by the static field (Fig. 2b) but also by varying the driving current.

## Discussion

**Figure 4:**
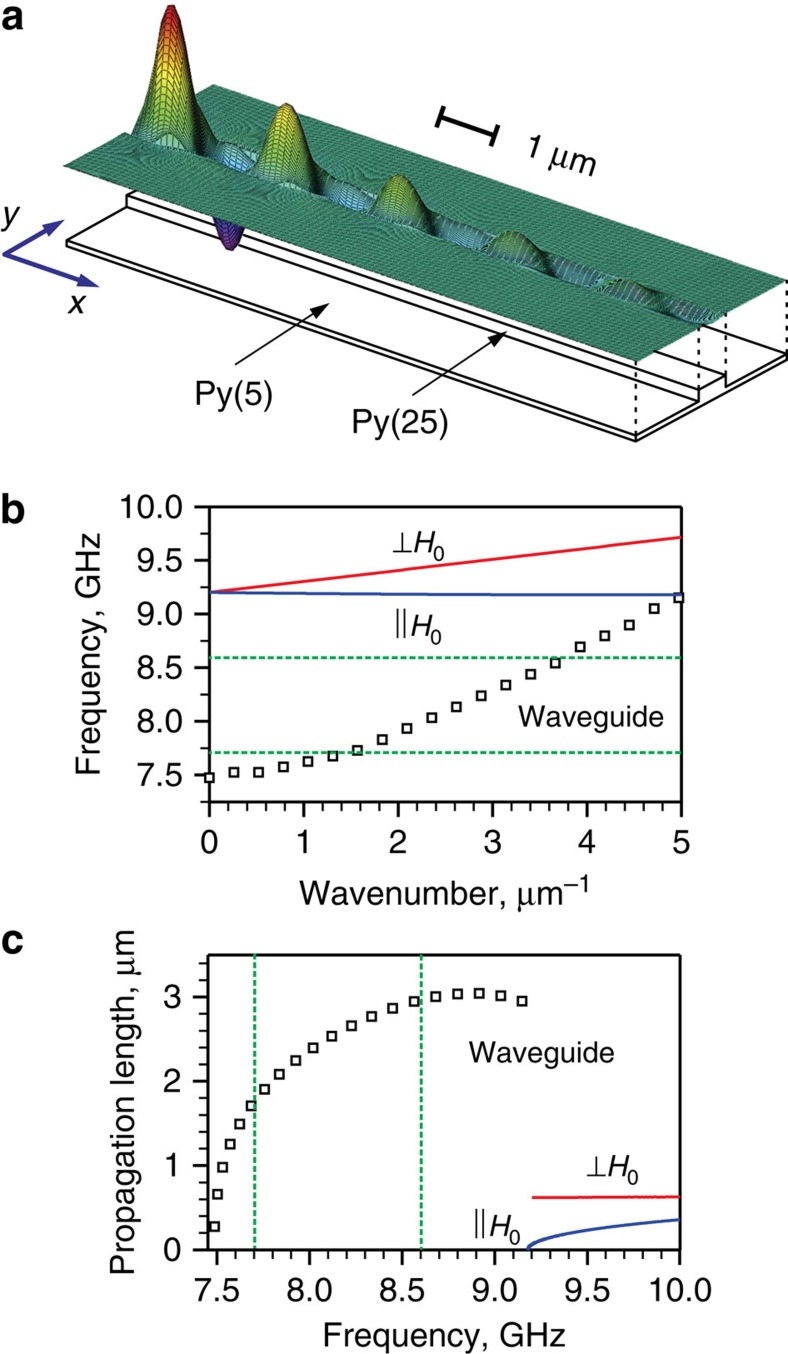
Micromagnetic simulations of the spin-wave propagation. (**a**) Snapshot of the out-of-plane component of the dynamic magnetization in a spin wave with the frequency of 8.6 GHz guided by the profiled-thickness waveguide. The size of the shown area is 3 μm in the direction transverse to the propagation and 10 μm parallel to it. (**b**) Calculated dispersion characteristics for spin waves guided by the waveguide (open squares) and for spin waves in an extended 5-nm-thick Py film propagating perpendicular (⊥*H*_0_) and parallel (||*H*_0_) to the direction of the static field. Horizontal dashed lines show the boundaries of the auto-oscillation frequency range of the spin-current oscillator. (**c**) Calculated frequency dependence of the spin-wave propagation length for the waveguide mode and for the waves in an extended film. Calculations were performed for *H*_0_=1,000 Oe.

To obtain insight into the mechanisms controlling the spin-wave propagation in the studied system, we performed micromagnetic simulations of the spin-wave dynamics in the profiled-thickness waveguide. Figure 4a shows a typical spatial map of the out-of-plane component of the dynamic magnetization obtained from the simulations. These data clearly show that the spin waves are well localized in the area with the increased thickness, in agreement with the experimental findings. By analysing the relation between the frequency and the wavelength obtained from the simulations, we determine the dispersion of spin waves in the waveguide (open squares in Fig. 4b). These results show that the lowest frequency of spin waves in the waveguide is 7.5 GHz, below the smallest auto-oscillation frequency of the NLSI oscillator, so that the waveguide supports propagation of spin waves at all frequencies within the auto-oscillation frequency range marked in Fig. 4b by the horizontal dashed lines. For comparison, we also show in Fig. 4b the dispersion curves for the waves in the extended film propagating perpendicular (⊥*H*_0_) and parallel (||*H*_0_) to the direction of the static magnetic field. The curves were calculated using the analytical spin-wave theory^35^. These data show that the dispersion spectrum in the extended film starts at ∼9.2 GHz. Above this frequency, the spin waves are no longer confined by the waveguide and instead propagate throughout the extended film. Thus, the total width of the confined-wave frequency band supported by the studied geometry of the profiled-film waveguide is ∼1.7 GHz.

Our simulations also allow us to calculate the propagation length of spin waves supported by the waveguide and the extended magnetic film (Fig. 4c). The results of calculations for the waveguide mode are in excellent quantitative agreement with the experiment (Fig. 3c). They also reproduce well the observed decrease of the propagation length with decreasing auto-oscillation frequency (Fig. 3c), which is probably caused by the reduction of the group velocity as the frequency approaches the bottom of the spin-wave dispersion at 7.5 GHz. This can be also seen from the decreasing slope of the dispersion curve in Fig. 4b.

Our analysis of the propagation characteristics indicates that by shifting the auto-oscillation range of the NLSI oscillator to larger frequencies, one can further increase the propagation length of the guided spin waves. Such frequency shift can be easily achieved by varying the geometry of the field-compensation elements (Fig. 1b), resulting in the variation of the dipolar magnetic field at the location of the auto-oscillator.

Finally, we discuss the power consumption and Joule heating in the proposed devices. The total resistance of the test devices, including the leads, is ∼20 Ω. The total consumed electrical power is then 1-2 mW within the used range of driving currents. As the devices perform all the functions necessary for the magnonic operation, ranging from the conversion of the dc current into microwave oscillations to generation of propagating spin waves, one can conclude that their power efficiency is superior to the magnonic systems that use spin-wave excitation using microwave currents generated by the traditional external microwave sources. Another advantage of the proposed devices lies in the weakness of their Joule heating. Heat flow simulations presented in the Supplementary Fig. 3 show that the temperature increase due to Joule heating does not exceed 7 K within the used range of currents. This value is significantly smaller than for the spin-current devices based on SHE^36^, owing to two important factors. First, the heat generation is low because of the high electrical conductivity of the thick current-carrying Cu layer. Second, this layer also exhibits a high thermal conductivity and thus plays an additional role of an effective heat sink.

In conclusion, we demonstrated that directionally propagating spin waves can be efficiently generated by using pure spin currents produced by the nonlocal spin-injection mechanism. The unique optically accessible layout of the studied devices enables direct observation of the current-induced magnetic oscillations and the emitted spin waves, and quantitative characterization of the device efficiency. Moreover, the geometry and the topography of the active magnetic layer in these devices can be easily modified, allowing complete control over the dynamic characteristics of the system. The obtained results open new perspectives for the future-generation electronics using electron spin degree of freedom for transmission and processing of information on the nanoscale.

## Methods

### Sample fabrication

An Au(65 nm) bottom electrode was fabricated on the sapphire substrate by e-beam lithography and sputtering. A circular Al(50 nm) mask with the diameter of 50 nm defined the point contact (PC). Ar ion milling was used to remove 22-nm-thick portion of Au layer everywhere, except for the area protected by the Al mask, followed by sputtering of undoped Si(22 nm). The insulating Si and the Al mask were removed from Au at the location of PC by oblique Ar ion milling and etching in a dilute solution of hydrofluoric acid (HF) in water, resulting in a flat surface of Au PC with height within ±5 nm relative to the surrounding Si, as tested by atomic-force microscopy. A Co_70_Fe_30_(8 nm)Cu(20 nm)Py(5 nm) layer covering the PC formed the top electrode, with an additional patterned Py(20 nm) layer defining the dipolar waveguide. The entire surface was coated with SiO_2_(50 nm), to protect the structure from oxidation.

### Magneto-optical measurements

Micro-focus BLS measurements were performed by focusing probing light with the wavelength of 532 nm produced by a continuous-wave single-frequency laser into a diffraction-limited spot. The analysis of the light scattered from magnetic oscillations was performed by using a six-pass Fabry–Perot interferometer TFP-1 (JRS Scientific Instruments, Switzerland). The obtained BLS intensity is proportional to the square of the amplitude of the dynamic magnetization at the location of the probing spot. Two-dimensional maps of the dynamic magnetization were recorded by rastering the spot over the surface of the sample using a closed-loop piezo-scanner. The spatial step size was 100 nm. The long-term spatial stability of better than 50 nm was achieved by using active feedback based on the custom-designed software. The estimated spatial resolution of the technique is 250 nm (ref. 7). All measurements were performed at room temperature.

### Micromagnetic simulations

The micromagnetic simulations were performed by using the software package MuMax3 (ref. 37). The computational domain with dimensions of 40 μm × 3 μm × 0.025 μm containing a 5-nm-thick Py film and a 500-nm-wide stripe-shaped area with the total thickness of 25 nm was discretized into 100 nm × 10 nm × 5 nm cells. The 100-nm cell dimension along the waveguide was found to be sufficient for accurate description of spin waves with the wavelengths of >1 μm studied in this work. This was verified by additional calculations using the cell size of 10 nm × 10 nm × 5 nm, which yielded similar results. The spin waves were excited by applying sinusoidal dynamic magnetic field with the amplitude of 1 Oe in the central area of the computational domain. Standard Gilbert damping constant of 0.01 (ref. 38) and the exchange stiffness of 1.3 × 10^−11^ J m^−1^ were used; the value of the saturation magnetization 4*πM*_0_=9.8 kG was independently determined from the BLS measurements of thermally excited spin waves.

### Calculations of the current distribution and the heat flow

Calculations were performed using a three-dimensional finite-element numerical model of the sample. The three-dimensional problem was numerically solved using COMSOL Multiphysics v5.0 software (COMSOL Inc.). The computational domain was discretized by using a tetrahedral mesh with the smallest element size of 0.025 nm in the area of the PC. Independently measured values of 2.5 × 10^6^, 1.6 × 10^7^ and 4 × 10^6^ S m^−1^ were used for the conductivities of Py, Cu and CoFe layers, respectively.

## Additional information

**How to cite this article:** Demidov, V. E. *et al*. Excitation of coherent propagating spin waves by pure spin currents. *Nat. Commun.* 7:10446 doi: 10.1038/ncomms10446 (2016).

## Supplementary Material

Supplementary Information Supplementary Figures 1-3
