# Supplementary material for: Excitation of coherent propagating spin waves by pure spin currents
Source: Nat Commun. 2016 Jan 28;7:10446. doi: 10.1038/ncomms10446 (PMC4738342; doi:10.1038/ncomms10446)
Supplement: Supplementary Information — Supplementary Figures 1-3 [file ncomms10446-s1.pdf]

Supplementary Figures

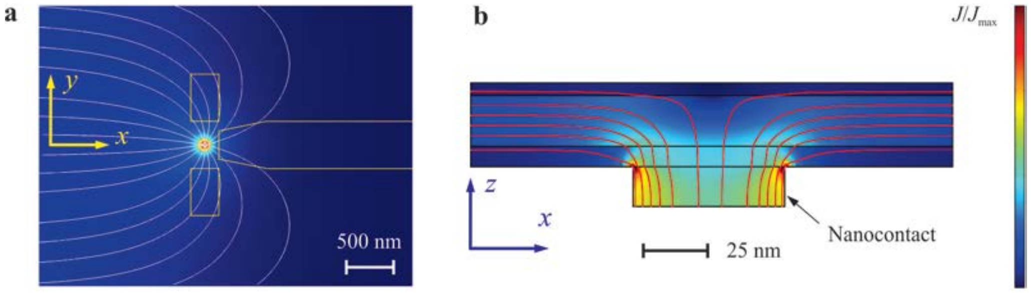

Supplementary Figure 1. Calculated spatial distributions of the density of the driving electrical current in the test devices. Color represents the magnitude of the current density, the curves are the streamlines of the current flow. Color scale: blue – 0, dark red – 1. **a,** Distribution of the current density in the plane of the current-carrying Cu layer. **b,** Distribution of the current density in the vertical cross-section along the *x* axis. The panel shows a zoom-in on the region close to the nanocontact. Note that the current flow is almost completely symmetric in the vicinity of the nanocontact, despite asymmetrical current injection apparent on the larger scale in panel **a**. As a result of this symmetry and of the absence of the vertical current flow through the top Py surface, the current in the Py layer vanishes right above the nanocontact.

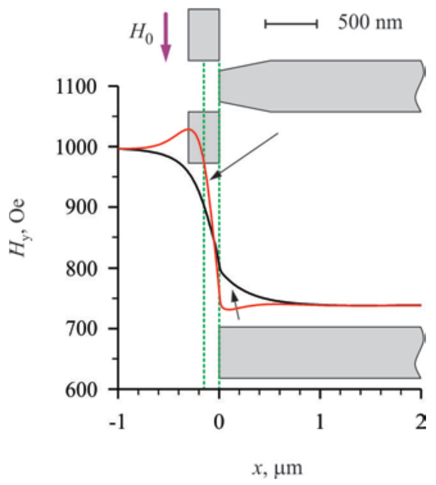

Supplementary Figure 2. Minimization of the dipolar-field effects. Calculated spatial profiles of the *y*-component of the internal static magnetic field for a simple rectangular-strip waveguide (black curve) and for the optimized profiled-film waveguide (red curve). Vertical dashed lines mark the positions of the waveguide edge (x=0) and the nanocontact (x=-150 nm). Calculations were performed at H_0=1000 Oe. In case of a simple waveguide, the internal field smoothly varies across the waveguide edge, resulting in two undesirable effects. First, the internal field significantly varies along the waveguide, increasing towards its edge. This causes a shift of the spectrum of propagating spin waves to higher frequencies, reducing coupling to the localized current-induced auto-oscillations. Second, because of the dipolar field of the waveguide, the static field is significantly reduced at the location of the nanocontact, causing a downshift of the auto-oscillation frequency. Both of these effects lead to the reduction of the frequency range for efficient emission of spin waves into the waveguide, which requires their spectral matching with the nano-oscillator. These effects are avoided in the optimized structure by tapering the waveguide to the width of 300 nm at the edge, and by adding two rectangular elements with dimensions of 500 nm by 300 nm, and 500 nm edge-to edge separation. These modifications enable a significantly more abrupt variation of the static internal field across the edge of the waveguide. The field becomes nearly uniform inside the waveguide, while abruptly increasing outside the waveguide to the magnitude very close to the applied external field H_0=1000 Oe.

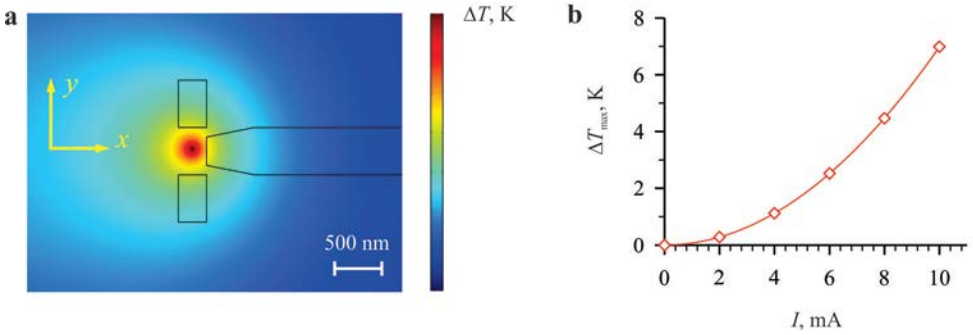

Supplementary Figure 3. Calculated increase of temperature caused by the Joule heating by the driving current. a, Spatial distribution of the current-induced temperature increase in the plane of the current-carrying Cu layer. The distribution was calculated for the maximum used current of 10 mA. Color scale: blue - 0, dark red – 7 K. **b,** Current dependence of the maximum temperature increase.
